# Validity of Digital Imaging of Fiber-Optic Transillumination in Caries Detection on Proximal Tooth Surfaces

**DOI:** 10.1155/2017/8289636

**Published:** 2017-10-01

**Authors:** Marja-Liisa Laitala, Liina Piipari, Noora Sämpi, Maria Korhonen, Paula Pesonen, Tiina Joensuu, Vuokko Anttonen

**Affiliations:** ^1^Research Unit of Oral Health Sciences, Department of Cariology, Endodontology and Paediatric Dentistry, University of Oulu, Oulu, Finland; ^2^Research Unit of Oral Health Sciences, University of Oulu, Oulu, Finland; ^3^Kuopio University Hospital, Kuopio, Finland; ^4^Medical Research Center, University of Oulu, Oulu University Hospital, Oulu, Finland

## Abstract

**Objective:**

The aim of our study was to evaluate the validity of the digital imaging fiber-optic transillumination (DIFOTI) method in comparison with clinical visual examination (CV) and bitewing (BW) radiography on detecting caries lesions on proximal surfaces of teeth.

**Materials and Methods:**

Proximal tooth surfaces of premolars and molars (*n* = 2,103) of 91 voluntary university students aged from 18 to 30 years were examined with CV, BW radiography, and the DIFOTI method.

**Results:**

DIFOTI detected more initial and manifested caries lesions compared with CV and BW. Of the analyzed tooth surfaces, 69.8% were classified as sound by DIFOTI, 80.3% by BW, and 91.6% by CV. Initial caries lesions were found in 21.2% of the surfaces by DIFOTI, in 14.1% by BW, and in 6.2% by CV, whereas the proportions for manifested dental caries lesions were 9.0%, 5.6%, and 2.2%, respectively. The interexaminer agreement regarding the DIFOTI findings between an experienced clinician and a fifth-year dental student was high: *κ* = 0.67 for initial and *κ* = 0.91 for manifested caries lesions.

**Conclusions:**

The noninvasive DIFOTI method seems to offer a potential tool for everyday clinical practice. In clinical use, DIFOTI finds well even initial caries lesions on proximal surfaces, thus providing an instrument for detecting lesions potential for arresting as well as for monitoring the outcome after preventive measures.

## 1. Introduction

Diagnosing caries lesions, especially initial lesions, is challenging [1, 2]. However, detection of caries lesions as early as possible is a cornerstone in a recent schema of caries controlling [3]. Diagnosing the disease in its early stages enables arresting the progress of initial lesions [3]. Visual-tactile has a long history as the most common diagnostic tool in caries detection. Currently, clinical visual examination (CV) including classifications of the progression and the depth of lesions [4, 5] as well as estimation of the lesion activity [6] is recommended. As a result of this improvement in caries detection protocol, the sensitivity of visual examination has improved [7, 8]. Conventional additional diagnostic methods for CV include bitewing (BW) radiography and fiber-optic transillumination (FOTI). Combined with BW, CV leads to more favorable diagnostic results than CV alone, especially with respect to proximal caries lesions [9–12]. BW images cannot be replaced by the FOTI method [13].

A need for additional tools for early caries detection is clear. During recent decades, research on caries detection has mainly focused on validating methods aiming to detect visually caries lesions of different degrees and evaluating early diagnostics tools [2, 14]. Digital imaging fiber-optic transillumination (DIFOTI) is among the most recent caries detection methods. In DIFOTI, the course of the near-infrared light beam is different in sound tissue compared to damaged tissue. With DIFOTI, the findings can be stored as digital images and displayed on a monitor. Fibers lead the light on the tooth surface and the tooth is transilluminated from both sides through a tip or a sensor in a plastic handgrip of the device. The tip has a tiny camera, and a digital image of the tooth is transmitted to the monitor online. Captured images can be saved in the database. The commercial application has been in use since the early 2000s [14], but scientific evidence on the clinical validity and clinical relevance of the DIFOTI method in caries detection is so far vague [15–19].

The aim of our study was to evaluate the validity of the DIFOTI method in caries detection on the proximal tooth surfaces in comparison with clinical visual examination and bitewing radiography, as well as its feasibility in association with work experience.

## 2. Materials and Methods

### 2.1. Participants

All 18–30-year-old university students who had reserved an appointment for a dental examination between October 2013 and February 2014 at the dental clinic of the Finnish Students' Health Service (FSHS) in Oulu, Finland, were invited to participate. Participation was voluntary, and altogether 137 participants were recruited. Of those, 91 participants had caries data on CV, BW radiographs, and DIFOTI images.

### 2.2. Methodology

Findings of CV, BW radiography, and DIFOTI were analyzed separately.

#### 2.2.1. Clinical Visual Examination

The clinical visual inspection was carried out in a fully equipped dental unit with light using a WHO probe and an oral mirror. Caries clinical staging was recorded by using ICDAS-classification (1–6) as follows: Score 0 = sound tooth surface, Score 1 = first visual change in enamel, Score 2 = distinct visual change in enamel, Score 3 = localised enamel breakdown, without visible dentine exposure, Score 4 = underlying dentine shadow, Score 5 = distinct cavity with visible dentine, and Score 6 = extensive cavity with visible dentine. In case of doubt dentists were advised to choose the higher option.

#### 2.2.2. Bitewing Radiographs and DIFOTI Images

Bitewing radiographs were taken according to the Finnish Current Care Guidelines for Controlling Dental Caries criteria only when clinically indicated (at least one caries lesion with dentin exposure or if the last BW radiographs had been taken more than three years earlier) [20]. All the radiographs were analyzed together by three of the authors, that is, experienced clinicians VA, M-LL, and TJ. Consensus was achieved on all the findings by the team.

A representative of the manufacturer provided education on the use of the DIFOTI device in hands-on sessions. The clinicians were advised to scan all the 1st and 2nd premolars and molars of the participants. A dental nurse at FSHS recorded the findings to the database according to the instructions of the device used. The images were saved for later analyses by using a specific programme offered by the manufacturer.

In the analyses, the distal and mesial surfaces of molars and premolars in BW and DIFOTI images were classified as follows: Score 0 = no caries; Score 1 = caries lesion in outer surface of the enamel; Score 2 = caries lesion extending into the inner enamel or dentoenamel-junction; Score 3 = dentinal caries lesion in the external half of dentin; Score 4 = deep dentinal caries lesion extending into the dentin half near the pulp. In the analyses, missing and failed images were analyzed separately and reported elsewhere.

To calculate the interexaminer agreement for DIFOTI, images of the 91 participants (altogether 2083 tooth surfaces) were analyzed by VA and a fifth-year dental student (NS) separately but at the same time to avoid bias caused by not being able to recognize the tooth or tooth surface correctly.

#### 2.2.3. Examiners

Before the present study, all the dentists (*n* = 7) working at the FSHS in Oulu were trained and calibrated by two of the authors (VA and M-LL) who had previous experience of introducing the protocol and training and calibrating examiners from several similar clinical studies. The training comprised PowerPoint presentations and hands-on in vitro training on caries detection with ICDAS criteria [5] to estimate caries lesion depth and activity. All the dentists participating in the clinical examinations were experienced. One dentist carried out all clinical examinations for one patient: the clinical visual inspection, BW radiographs, and DIFOTI scanning. A dental nurse recorded the findings on structured forms.

The calibration of the examiners was carried out using ICDAS criteria with caries activity assessment on extracted teeth. For the purposes of quality assurance of the clinical examination, the gold standard (M-LL) performed repeated clinical examinations to the patients of each examiner.

### 2.3. Statistical Analyses

The data were described as frequencies and proportions. To evaluate the findings regarding different tooth groups (upper and lower premolars and molars), cross tabulation was used. Detection rate (sensitivity) and specificity for DIFOTI were calculated by using BW radiographs as the gold standard method; the cut-off points used were initial caries lesions (lesions clinically restricted to enamel/without dentin exposure) and manifested caries lesions (lesions with clinical dentin exposure). Additionally, DIFOTI findings were evaluated by using CV as the gold standard, and CV was compared with BW.

To investigate the interexaminer agreement of the findings between the two examiners analyzing DIFOTI images, kappa values were calculated. For the analyses, SPSS (version 22.0, SPSS, Inc., Chicago, IL, USA) was used.

### 2.4. Ethical Considerations

The Regional Ethics Committee of the Northern Ostrobothnia Hospital District (EETTMK: 102/2013) and the board of FSHS gave their approval for the study. Participation was voluntary, and all the participants gave their written consent. Data were collected and analyzed without personal IDs.

## 3. Results

Altogether 1162 teeth were analyzed. Of those, 591 were molars (292 upper, 299 lower) and 571 premolars (293 upper and 278 lower, resp.). DIFOTI detected more initial and manifested caries lesions compared with the CV and BW methods (Table 1). Of the analyzed proximal tooth surfaces of premolars and molars (*n* = 2,103), about 90% (*n* = 1,927) were classified as sound by CV, 80% (*n* = 1,688) by BW, and 70% (*n* = 1,468) by DIFOTI. Initial caries lesions were found in one-fifth of the surfaces by DIFOTI; the proportion was more than three times higher when compared with findings by CV and almost double when compared with BW. DIFOTI found manifested caries lesions in 9% of the tooth surfaces; respective figures for CV and BW were 2% and 6%.

With respect to the tooth group, the majority of the manifested caries lesions detected by using the CV and BW methods were found in the upper premolars (CV: *n* = 25, BW: *n* = 48, DIFOTI: *n* = 57), whereas by DIFOTI, the majority of the manifested lesions were in the upper molars (CV: *n* = 9, BW: *n* = 36, and DIFOTI: *n* = 59). The number of initial caries lesions in the upper molars (*n* = 142) detected by using DIFOTI was the double of the number detected in the lower premolars (*n* = 71). The corresponding figures using DIFOTI for upper premolars were *n* = 116 and for lower premolars *n* = 117. For CV and BW, the distributions of the initial caries findings were more even: CV upper premolars *n* = 36, lower premolars *n* = 19, upper molars *n* = 38, and lower molars *n* = 37 and BW upper premolars *n* = 75, lower premolars *n* = 56, upper molars *n* = 85, and lower premolars *n* = 82, respectively. However, with all three methods lower premolar teeth showed higher number of sound surfaces (Table 1).

Comparing DIFOTI findings with BW findings, the detection rate was 54.2% when using initial caries lesions as the cut-off point and 46.2% when using manifested caries lesions as the cut-off point. Specificity was 75.7% for initial caries lesions and 93.2% for manifested caries lesions (Table 2(a)). When comparing DIFOTI findings with CV findings, the detection rate was 55.1% and specificity 72.1% with initial caries lesions as the cut-off point, while the respective figures for manifested caries lesions were 47.8% and 93.0%, respectively (Table 2(b)).

Of the methods used, CV found the least initial and manifested caries lesions (Table 2(c)), the difference being more distinct between CV and DIFOTI compared with CV and BW. The interexaminer agreement concerning DIFOTI findings between the experienced clinician and the fifth-year dental student was high (0.67). When using manifested caries as the cut-off point, the agreement was excellent (0.91) (Table 3).

## 4. Discussion

In our clinical study, the digital fiber-optic transillumination method, DIFOTI, detected significantly more initial and manifested caries lesions on proximal surfaces compared with CV and BW radiography. Furthermore, the agreement in caries detection was not dependent on the work experience.

Research on the validity of DIFOTI in caries detection is limited. Our results support the outcomes of two recent clinical studies, which concluded that DIFOTI may reduce the need for BW radiography when detecting caries on proximal tooth surfaces [18, 19]. In their* in vitro* study with histological validation, Astvaldsdóttir et al. [16] reported outcomes similar to ours: DIFOTI was superior in detecting initial caries lesions compared with both CV and BW radiography.

Value of BW radiography has been widely studied clinically. Hietala-Lenkkeri et al. [10] compared CV and BW and reported BW to be beneficial for 14-year-olds in a population with low caries prevalence. In the study of Poorterman et al. [9], the number of surfaces in need of restorative treatment (due to caries or an inadequate restoration) was doubled based on an additional BW examination compared with visual examination alone, BW being most beneficial among 17-year-olds. However, in their study, the same was not true for the 14-year-olds. Our results are somewhat similar, but the DIFOTI method is clearly superior to BW radiography.

Gold standard is always a problem in* in vivo* studies investigating the validity of caries detection methods. The low values concerning sensitivity and specificity of DIFOTI are explained by the fact that BW radiography and CV were used as the gold standard, a “true finding,” for DIFOTI. Here, the “false positive” findings are most likely true additional positive findings. DIFOTI detected significantly more initial caries lesions and more manifested lesions than BW. When using CV as the gold standard, the respective figures were even higher.

In an* in vivo* study, a 2-day temporary separation of the teeth increases the accuracy of the clinical visual examination. This was not possible in our study design and can be considered as a limitation. It must be kept in mind that the quality of DIFOTI images and also of BW radiographs can influence the outcome of caries detection. Under- and overestimation in either case may have caused bias. However, the influence of the limitations on the methods can only be speculated. Despite these uncertainties, the good interexaminer agreement of DIFOTI also supports the validity of the method. Nevertheless, longitudinal studies would be valuable in this respect.

Our study population comprised young adults with fairly low caries prevalence and in most cases only a few restorations. The DIFOTI method easily detects even tooth-colored restorations, which is a benefit. On the other hand, large composite and amalgam restorations may hinder secondary caries detection. In the present study, the study population was appropriate for this type of a study focusing on comparing methods. The study group could have been larger, but it is in line with a recent clinical study on the topic with similar outcomes [19].

While analyzing the images, we found that DIFOTI reveals not only carious lesions but also macro- and microfractures. This provides an additional benefit to use DIFOTI. In their recent systematic review, Innes and Schwendicke [21] found noteworthy heterogeneity in the treatment decisions and restorative thresholds among clinicians; a significant proportion of the caries lesions which should be noninvasively treated are intervened restoratively. Good-quality DIFOTI images show clearly the depth of the caries lesions in the tissue, which helps the clinician to decide whether to choose a noninvasive or invasive treatment. This may reduce unnecessary restorations and give a possibility of implementing successful preventive measures. Because DIFOTI images show only one tooth at a time, the images provide more detailed information than BW radiography, provided that the DIFOTI images are of high quality. The quality of the DIFOTI images and challenges in the technique should be recognized and research of this topic would be valuable.

Compared to FOTI, the advantage of the DIFOTI method is that the image can be stored and that it is easy to use for monitoring lesion progression and effects of preventive measures. The real-time view is also useful to illustrate the condition of the dentition tooth by tooth, not only to the dentist but also to the patient. It could be an easy and efficient tool to motivate patients towards better home care. So far, we have not found any clinical studies comparing these two methods.

The most significant advantage of the method is that it does not cause any extra radiation exposure to the patient. Therefore, it is a safe option even when radiographs are susceptive or contraindicated. In addition, it can be speculated that DIFOTI may cause less discomfort for the patient than BW radiography, when the patients sometimes find the BW film plate uncomfortable. The present DIFOTI device (DIAGNOCam, KaVO, Biberach, Germany) has two sizes of sensors, and selecting the right size makes imaging more comfortable to the patient. Being portable, the device can be shared by several dentists. When comparing the advantages of DIFOTI with BW radiography, also costs and other economic aspects have to be considered. This would be an interesting topic for the future research.

There are some crucial aspects to consider when using DIFOTI. The labelling of the tooth in the saved DIFOTI image must be exact and clear to ensure the right diagnosis and to enable consultation between clinicians. It is impossible for other users compared to the examiner to analyze DIFOTI images later, if the tooth is not properly labelled. Even if the tooth can be recognized, determining the right surface is challenging. This aspect must be kept in mind when evaluating the reliability of the DIFOTI method. Not being able to identify the tooth/tooth surface may cause bias when comparing the outcome. In our study, only the images which were properly labelled and standardized as for tooth surfaces were included in the analyses. Scanning with the DIFOTI device can also be challenging. Proper and thorough training on the use of the DIFOTI device is necessary to assure good image quality.

## 5. Conclusions

The noninvasive DIFOTI method seems to offer a potential tool in everyday clinical practice for the detection and assessment of caries lesions in proximal surfaces. Specifically, young populations with fairly low caries prevalence seem to benefit from the use of DIFOTI, because the method is at its best when there are none or just a few fillings in the dentition.

In clinical use, DIFOTI finds well initial caries lesions, thus providing an instrument for detecting lesions potential for arresting as well as for monitoring the outcome after preventive measures. It can also be useful in motivating the patient towards good oral self-care.

## Figures and Tables

**Table 1:** Distribution of surfaces with different stages of dental caries. Findings of the clinical visual method (CV), bitewing (BW) radiographs, and fiber-optic transillumination (DIFOTI) are presented separately for upper and lower premolars and molars. The total number of analyzed tooth surfaces was *n* = 2,103.

Proportions (%) of tooth surfaces with different stages of caries lesions									
	Sound			Initial			Manifested		
	CV	BW	DIFOTI	CV	BW	DIFOTI	CV	BW	DIFOTI
Upper premolars *n* = 566	89.2	78.3	69.4	6.4	13.3	20.5	4.4	8.5	10.1
Lower premolars *n* = 521	95.6	87.3	82.1	3.6	10.7	13.6	0.8	1.9	3.6
Upper molars *n* = 507	90.7	76.1	60.4	7.5	16.8	28.0	1.8	7.1	11.6
Lower molars *n* = 509	91.2	79.4	66.4	7.3	16.1	23.0	1.6	4.5	10.6
Total *n* = 2,103	91.6	80.3	69.8	6.2	14.1	21.2	2.2	5.6	9.0

**Table:** (a) DIFOTI versus BW

	DIFOTI				
		Sound	Initial	Manifested	Total (%)
BW	Sound	1,278 (75.5)	329 (19.5)	81 (4.8)	1,688 (80.3)
	Initial	157 (52.7)	87 (29.2)	54 (18.1)	298 (14.2)
	Manifested	33 (28.2)	30 (25.6)	54 (46.2)	117 (5.6)
	Total (%)	1,468 (69.8)	446 (21.2)	189 (9.9)	2,103

**Table:** (b) DIFOTI versus CV

	DIFOTI				
		Sound	Initial	Manifested	Total (%)
CV	Sound	1,389 (72.1)	391 (20.3)	147 (7.6)	1,927 (91.6)
	Initial	68 (52.3)	42 (32.3)	20 (15.4)	130 (6.2)
	Manifested	11 (23.9)	13 (28.3)	22 (47.8)	46 (2.2)
	Total (%)	1,468 (69.8)	446 (21.2)	189 (9.9)	2,103

**Table:** (c) CV versus BW

	CV				
		Sound	Initial	Manifested	Total (%)
BW	Sound	1,595 (94.5)	75 (4.4)	18 (1.1)	1,688 (80.3)
	Initial	251 (84.2)	39 (13.1)	8 (2.7)	298 (14.2)
	Manifested	81 (69.2)	16 (13.7)	20 (17.1)	117 (5.6)
	Total (%)	1,927 (91.6)	130 (6.2)	46 (2.2)	2,103

**Table 3:** Findings of fiber-optic transillumination (DIFOTI) images by a fifth-year dental student and an experienced clinician; numbers and proportions of tooth surfaces.

Numbers and proportions (%) of tooth surfaces with different stages of caries lesions					
	DIFOTI (fifth-year dental student)				
		Sound	Initial	Manifested	Total (%)
DIFOTI (experienced clinician)	Sound	1,308	106	12	1,426 (68.5)
	Initial	132	278	32	442 (21.2)
	Manifested	15	56	144	215 (10.3)
	Total (%)	1,455 (69.8)	440 (21.2)	188 (9.0)	2,083 (100.0)

## References

[1] Baelum V. (2010). What is an appropriate caries diagnosis?. *Acta odontologica Scandinavica*.

[2] Twetman S., Axelsson S., Dahlén G. (2013). Adjunct methods for caries detection: A systematic review of literature. *Acta Odontologica Scandinavica*.

[3] Schulte A. G., Pitts N. B., Huysmans M. C. D. N. J. M., Splieth C., Buchalla W. (2011). European core curriculum in cariology for undergraduate dental students. *Caries Research*.

[4] Ekstrand K. R., Bruun G., Bruun M. (1998). Plaque and gingival status as indicators for caries progression on approximal surfaces. *Caries Research*.

[5] Pitts N. (2004). ‘ICDAS’—An international system for caries detection and assessment being developed to facilitate caries epidemiology, research and appropriate clinical management. *Community Dental Health*.

[6] Nyvad B., Machiulskiene V., Baelum V. (2003). Construct and predictive validity of clinical caries diagnostic criteria assessing lesion activity. *Journal of Dental Research*.

[7] Ismail A. I., Sohn W., Tellez M. (2007). The International Caries Detection and Assessment System (ICDAS): an integrated system for measuring dental caries. *Community Dentistry and Oral Epidemiology*.

[8] Jablonski-Momeni A., Stachniss V., Ricketts D. N., Heinzel-Gutenbrunner M., Pieper K. (2008). Reproducibility and accuracy of the ICDAS-II for detection of occlusal caries in vitro. *Caries Research*.

[9] Poorterman J. H. G., Aartman I. H. A., Kieft J. A., Kalsbeek H. (2000). Value of bite-wing radiographs in a clinical epidemiological study and their effect on the dmfs index. *Caries Research*.

[10] Hietala-Lenkkeri A.-M., Tolvanen M., Alanen P., Pienihäkkinen K. (2014). The additional information of bitewing radiographs in the detection of established or severe dentinal decay in 14-year olds: A cross-sectional study in low-caries population. *The Scientific World Journal*.

[11] Wenzel A. (2014). Radiographic display of carious lesions and cavitation in approximal surfaces: Advantages and drawbacks of conventional and advanced modalities. *Acta Odontologica Scandinavica*.

[12] Gimenez T., Piovesan C., Braga M. M. (2015). Clinical relevance of studies on the accuracy of visual inspection for detecting caries lesions: A systematic review. *Caries Research*.

[13] Vaarkamp J., Ten Bosch J. J., Verdonschot E. H., Bronkhorst E. M. (2000). The real performance of bitewing radiography and fiber-optic transillumination in approximal caries diagnosis. *Journal of Dental Research*.

[14] Stookey G. K., González-Cabezas C. (2001). Emerging methods of caries diagnosis. *Journal of Dental Education*.

[15] Bin-Shuwaish M., Yaman P., Dennison J., Neiva G. (2008). The correlation of DIFOTI to clinical and radiographic images in class II carious lesions. *Journal of the American Dental Association*.

[16] Astvaldsdóttir Á., Åhlund K., Holbrook W. P., De Verdier B., Tranæus S. (2012). Approximal caries detection by DIFOTI: In vitro comparison of diagnostic accuracy/efficacy with film and digital radiography. *International Journal of Dentistry*.

[17] Abdelaziz M., Krejci I. (2015). DIAGNOcam--a Near Infrared Digital Imaging Transillumination (NIDIT) technology. *The international journal of esthetic dentistry*.

[18] Kühnisch J., Söchtig F., Pitchika V. (2016). In vivo validation of near-infrared light transillumination for interproximal dentin caries detection. *Clinical Oral Investigations*.

[19] Lara-Capi C., Cagetti M. G., Lingström P. (2017). Digital transillumination in caries detection. *Dentomaxillofacial Radiology*.

[20] Finnish Current Care Guidelines for Controlling Dental Caries Current Care Guidelines. http://www.kaypahoito.fi/web/english/guidelineabstracts/guideline?id=ccs00105/.

[21] Innes N., Schwendicke F. (2017). Restorative thresholds for carious lesions: systematic review and meta-analysis. *Journal of Dental Research*.

